# Metabolomics reveals the mechanisms for the cardiotoxicity of Pinelliae Rhizoma and the toxicity-reducing effect of processing

**DOI:** 10.1038/srep34692

**Published:** 2016-10-04

**Authors:** Tao Su, Yong Tan, Man-Shan Tsui, Hua Yi, Xiu-Qiong Fu, Ting Li, Chi Leung Chan, Hui Guo, Ya-Xi Li, Pei-Li Zhu, Anfernee Kai Wing Tse, Hui Cao, Ai-Ping Lu, Zhi-Ling Yu

**Affiliations:** 1 Consun Chinese Medicines Research Centre for Renal Diseases, School of Chinese Medicine, Hong Kong Baptist University, Kowloon Tong, Hong Kong, China; 2 Institute of Basic Research in Clinical Medicine, China Academy of Chinese Medical Science, Beijing, China; 3 Department of Pathology, Caritas Medical Center, Hong Kong, China; 4 Department of Pathology, Guangzhou University of Chinese Medicine, China; 5 Institute of Integrated Bioinfomedicine & Translational Science, HKBU Shenzhen Research Institute and Continuing Education, Shenzhen, China; 6 College of Pharmacy, Jinan University, Guangzhou, China

## Abstract

Pinelliae Rhizoma (PR) is a commonly used Chinese medicinal herb, but it has been frequently reported about its toxicity. According to the traditional Chinese medicine theory, processing can reduce the toxicity of the herbs. Here, we aim to determine if processing reduces the toxicity of raw PR, and to explore the underlying mechanisms of raw PR-induced toxicities and the toxicity-reducing effect of processing. Biochemical and histopathological approaches were used to evaluate the toxicities of raw and processed PR. Rat serum metabolites were analyzed by LC-TOF-MS. Ingenuity pathway analysis of the metabolomics data highlighted the biological pathways and network functions involved in raw PR-induced toxicities and the toxicity-reducing effect of processing, which were verified by molecular approaches. Results showed that raw PR caused cardiotoxicity, and processing reduced the toxicity. Inhibition of mTOR signaling and activation of the TGF-β pathway contributed to raw PR-induced cardiotoxicity, and free radical scavenging might be responsible for the toxicity-reducing effect of processing. Our data shed new light on the mechanisms of raw PR-induced cardiotoxicity and the toxicity-reducing effect of processing. This study provides scientific justifications for the traditional processing theory of PR, and should help in optimizing the processing protocol and clinical combinational application of PR.

Pinelliae Rhizoma (PR), the dried tuber of *Pinellia ternata* (Thunb.) Breit., is a traditional Chinese medicinal herb^1^. It was first recorded in *Shen Nong Ben Cao Jing* (Shen Nong’s herbal classic), a book of 2,000 years ago. PR is commonly used in traditional Chinese medicine (TCM) prescriptions to manage cough, phlegm, vomiting and cancer^2^,^3^. Chemical studies revealed that PR contains alkaloids, organic acids, proteins, *etc*^4^,^5^. Pharmacological studies showed that PR has antitussive, antiemetic, expectorant and antitumor properties^6^,^7^,^8^. PR also has toxicities. In animals, the powder or extract of raw PR demonstrates acute toxicity, and excessive or long-term use of this herb can cause organ injuries^9^,^10^. In addition, raw PR has irritant, teratogenic, carcinogenic, mutagenic and reproductive toxicities^2^,^3^. Needle-like calcium oxalate crystals, lectin and protocatechualdehyde have been considered as the possible toxic substances of PR^11^,^12^,^13^. While, the mechanisms of raw PR-induced toxicity are still not fully understood.

Chinese medicinal materials (CMMs) need to be processed to become decoction pieces before they are prescribed in the clinic or used for producing proprietary drugs. According to the TCM theory, processing can reduce the toxicity of the herbs^14^. As recorded in Chinese Pharmacopoeia (CP)^1^, PR has four processed products including raw PR, Pinelliae Rhizoma Praeparatum cum Alumine (PRPA), Pinelliae Rhizoma Praeparatum (PRP) and Pinelliae Rhizoma Praeparatum cum Zingibere et Alumine (PRZA). PRZA is the product of raw PR processed with ginger juice and alumen. Previous studies demonstrated that PRZA was less toxic than raw PR in mice^15^,^16^. Processing caused structural changes of the toxic substances, e.g. needle-like calcium oxalate crystals, have been regarded as the toxicity-reducing mechanisms^15^. It has also been suggested that a compound gingerol from ginger juice can effectively inhibit raw PR-induced inflammation^17^. Nevertheless, up to the present, the underlying toxicity-reducing mechanisms of processing are uncertain.

Because of the multiple chemical components and the multi-target nature of Chinese medical herbs^18^, using conventional research approaches such as biochemical and histological analyses to elucidate the mechanisms for herbal toxicities and the toxicity-reducing effect of processing have limitations. Metabolomics, a systematic approach for analyzing the small-molecule metabolites (MW < 1 kd) using various analytical methods, and for figuring out the biological implications of the metabolites using bioinformatics means, has emerged as a powerful approach for this kind of studies^19^,^20^. In this study, we explored the mechanisms of raw PR-induced toxicities and the toxicity-reducing effect of processing using the metabolomics approach.

## Results

### General observations

**Figure 1:**
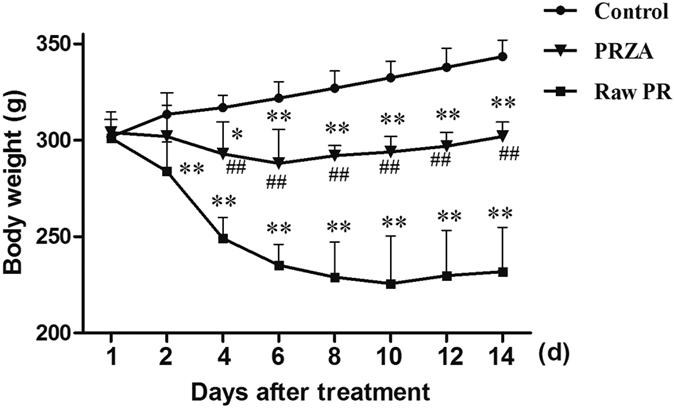
Body weights of vehicle-, raw PR- and PRZA-treated rats. *p < 0.05, **p < 0.01 *vs.* control; ^##^*p* < 0.01 *vs*. Raw PR group.

Compared with the control group, raw PR group showed a dramatic body weight loss from day 2 (*p* < 0.01); PRZA group appeared body weight loss from day 4 (*p* < 0.05) and the severity was significantly less than that of raw PR group (*p* < 0.01) (Fig. 1). In addition, we observed that raw PR- but not PRZA-treated rats suffered from diarrhea, and showed less physical activity.

### Biochemical changes

**Figure 2:**
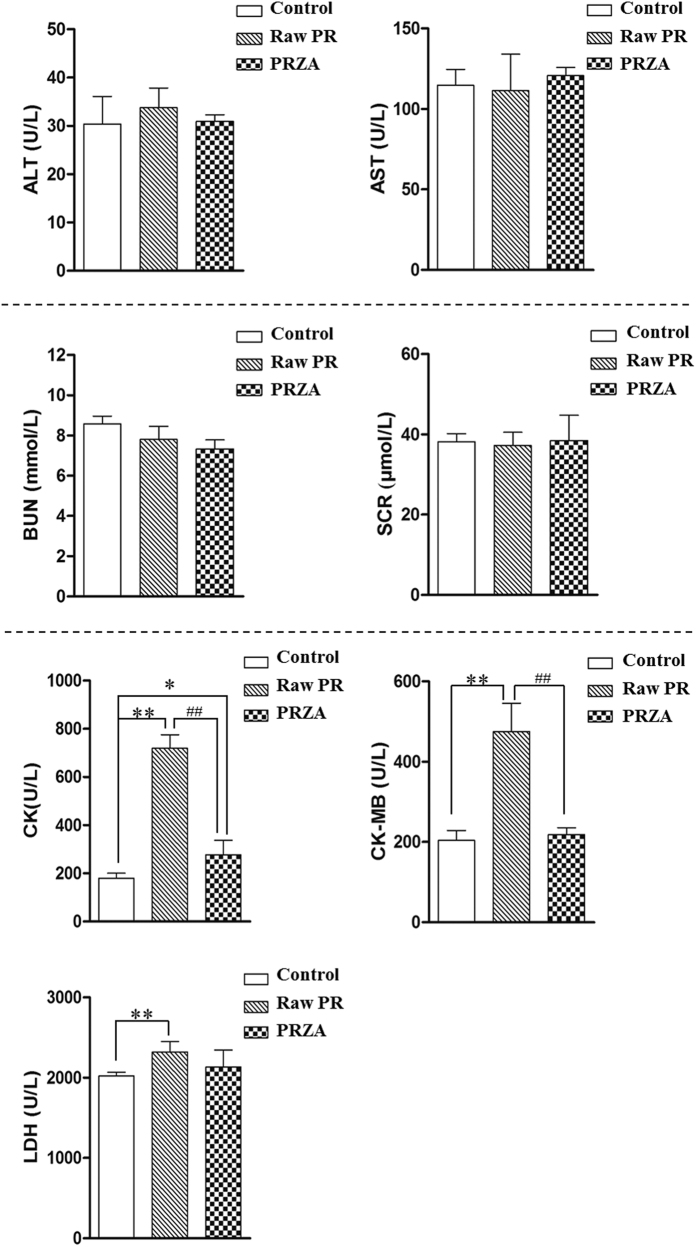
Biochemical parameters in the serum of vehicle-, raw PR- and PRZA-treated rats. *p < 0.05, **p < 0.01 *vs.* control; ^#^*p* < 0.05, ^##^*p* < 0.01 *vs.* Raw PR.

The serum levels of CK, CK-MB and LDH in raw PR group were all significantly higher than that in control group (*p* < 0.01) (Fig. 2), indicating that raw PR caused myocardial injury and myocardial infarction. Interestingly, there was no significant difference in the levels of CK-MB and LDH between vehicle- and PRZA-treated rats; compared with the vehicle, PRZA only increased the serum CK level (*p* < 0.05). Both raw PR and PRZA did not significantly affect the serum levels of ALT, AST, BUN and SCR (ALT and AST are markers of hepatic damage; BUN and SCR are markers of renal damage). These results indicated that raw PR caused cardiotoxicity, and PRZA was less toxic than raw PR. No obvious liver and kidney toxicities were observed in both raw PR- and PRZA-treated rats.

### Histopathological changes

**Figure 3:**
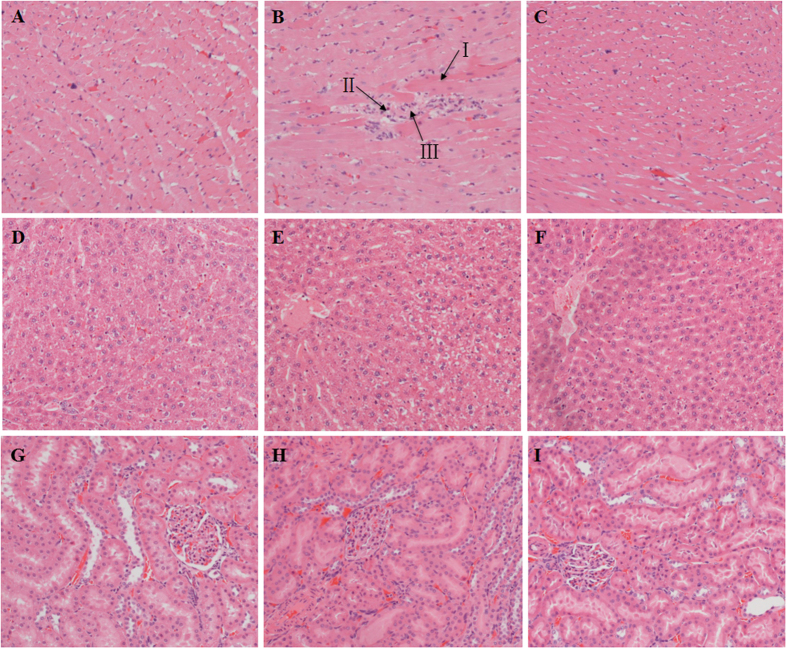
Histopathological examinations of heart, liver, kidney tissues in vehicle-, raw PR- and PRZA-treated rats, H&E staining, 100×. (**A**) Heart tissue of control group: normal myocardial fibers in longitudinal section featuring central nuclei and syncytial arrangement of the fibers; (**B**) Heart tissue of raw PR group: myocardial fibers with loss of cross striations, not clearly visible nuclei, and inflammatory infiltration; (**C**) Heart tissue of PRZA group: the histopathological changes were milder than raw PR-treated group; (**D**) Liver tissue of control group; (**E**) Liver tissue of raw PR group; (**F**) Liver tissue of PRZA group; (**G**) Kidney tissue of control group; (**H**) Kidney tissue of raw PR group; (**I**) Kidney tissue of PRZA group. (**D–I**) Liver and kidney sections of both PR and PRZA groups showed no abnormalities. I. Myocardiocyte necrosis; II. Myocardiocyte rupture; III. Inflammatory infiltration.

Histopathological damages were observed in the heart tissues of raw PR-treated rats with rupture and necrosis of the cardiomyocytes, and inflammatory cells infiltration, while, the damages were relatively mild in PRZA-treated rats (Fig. 3C). Liver and kidney sections of both PR and PRZA groups showed no abnormalities (Fig. 3D–I). These results further suggested that raw PR caused cardiotoxicity, and processing reduced the toxicity.

### Repeatability and stability of the LC-MS method

Six extracts from a random serum sample were continuously injected to the LC-MS system to evaluate the repeatability of the analytical method. Four common extracted ion chromatograms (EICs) shared by these injections were selected according to their different chemical polarities and m/z values. The relative standard derivations (RSDs) of these peaks were 4.04–14.51% for peak areas and 0.03–0.98% for retention times. These results indicated that our established LC-MS method was repeatable.

The LC-MS system stability for the large-scale samples analyses was demonstrated by the test of pooled QC samples. The peak areas, retention times and mass accuracies of four selected EICs in four QC samples showed good system stability. RSDs of the four peaks were 4.84–14.08% for peak areas, 0.02–1.01% for retention times, and 0.12 × 10–04%–0.87 × 10–04% for mass accuracies. These data indicated that the LC-MS system was stable.

### Identification of differential metabolites

**Figure 4:**
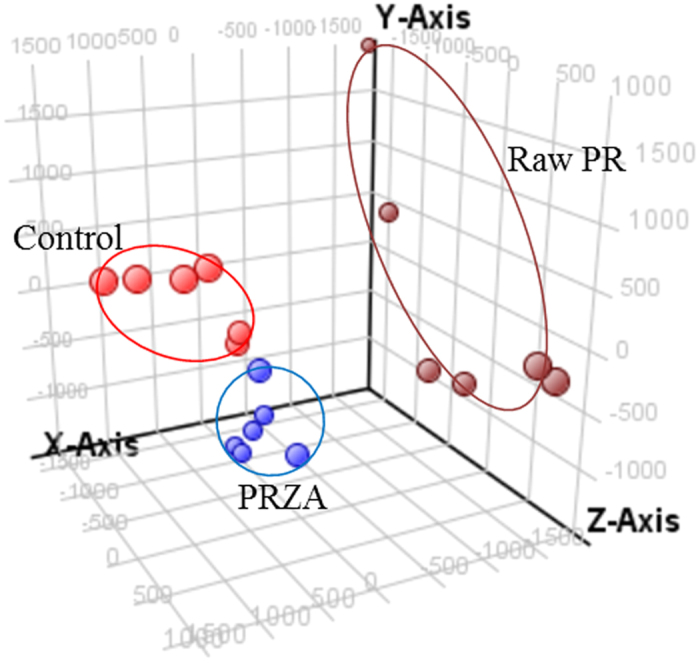
Results of the multiple pattern recognition of the serum metabolites in control, raw PR and PRZA groups at the time point of day 14 . (red 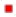) Control group, (brown 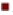) Raw PR group, (blue 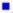) PRZA group.

Typical base peak intensity (BPI) chromatograms of the serum samples were obtained for vehicle-, raw PR- and PRZA-treated rats (Supplementary Fig. 5). Based on the metabolic differences among the three groups, we used PCA to classify the differential metabolic phenotypes. PCA scores (PC1: 21%, PC2: 14.8%, PC3: 13.4%) showed that the three groups were obviously clustered (Fig. 4), suggesting that treatment with raw PR/PRZA caused endogenous metabolite changes compared to the vehicle, and the metabolite changes are different between raw PR and PRZA groups. Multiple pattern recognition method of partial least squares discriminant analysis (PLS-DA) was adopted on the basis of the metabolic changes in three groups as revealed by BPI chromatograms. As shown in PLS-DA scores plot (Supplementary Fig. 6, R^2^X = 0.138, R^2^Y = 0.937, Q^2^ = 0.753), there was obvious separation among the control, raw PR and PRZA groups. Top 200 significant ions were selected for metabolite identification. Among them, 10 significantly altered metabolites were identified and were shown in Table 1.

Seven and eight significantly altered metabolites were identified in the serum samples of raw PR- and PRZA-treated rats, respectively (Table 1). Proline, dihydrouracil, dihydrosphingosine 1-phosphate (dhS1P) and 2-keto-4-methylthiobutyric acid (KMTB) were up-regulated, and saccharopine was down-regulated in both raw PR and PRZA groups. Leucine and 5-hydroxytryptamine (5-HT) were only up-regulated in raw PR group, while kynurenine, p-aminobenzoic acid (PABA) and tyrosine were only up-regulated in PRZA group.

### Metabolic pathway and network function analyses with IPA

**Figure 5:**
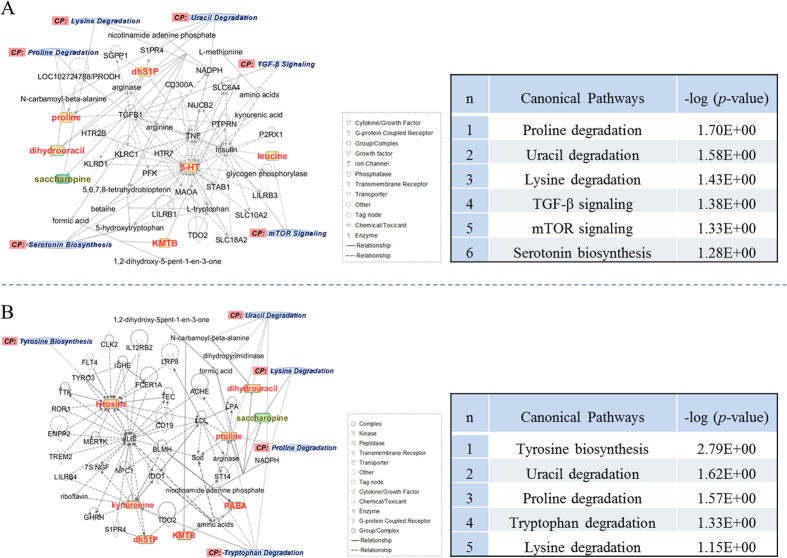
Molecular networks and the canonical pathways in the serum of raw PR- and PRZA-treated rats. (**A**) Raw PR group; (**B**) PRZA group. The network was gained by overlapping raw PR- or PRZA-treated group’s data to control group’s data. Metabolites are represented as nodes, and the biological relationship between two nodes is represented as a line. The colored symbols represent metabolites that occurring in the tested data, while the transparent entries are molecules from the Ingenuity Knowledge Database. Orange symbols represent up-regulated metabolites; green symbols represent down-regulated metabolites. Solid lines between molecules indicate a direct physical relationship between molecules; dotted lines indicate indirect functional relationships.

To further understand the correlation between the candidate metabolites and the biological association networks, we performed bioinformatics analysis using the IPA software. A network was built based on the seven differential metabolites identified in the serum samples of raw PR-treated rats. The established network functions included amino acid metabolism, lipid metabolism, small molecule biochemistry, cell-to-cell signaling and interaction, as well as vitamin and mineral metabolisms. The canonical pathways included proline, uracil and lysine degradations, the TGF-β and mTOR signaling pathways, as well as serotonin biosynthesis (Fig. 5A). Similarly, we have also mapped the metabolic network by using the eight differential metabolites identified in the serum samples of PRZA-treated rats (Fig. 5B). The established network functions include amino acid metabolism, post-translational modification, lipid metabolism, small molecule biochemistry, and free radical scavenging. The canonical pathways were tyrosine biosynthesis, and proline, uracil, tryptophan and lysine degradations.

Previous studies suggested that leucine up-regulation could activate mTOR signaling^21^,^22^, and excessive activation or inhibition of mTOR signaling could induce cardiac disorders^23^,^24^; 5-HT up-regulation might elevate TGF-β1 expression and activity, which could induce significant cardiovascular adverse effects, e.g., cardiac arrhythmias^25^ and cardiac valve abnormalities^26^,^27^; kynurenine, PABA and tyrosine have been reported to have free radical scavenging properties^28^,^29^,^30^. Therefore, we speculated that two highlighted pathways, the mTOR and TGF-β signaling pathways may be involved in raw PR-induced cardiotoxicity; and the predicated molecular network function, free radical scavenging may be responsible for the toxicity-reducing effect of processing.

### Verification of the mechanisms of raw PR-induced cardiotoxicity and the toxicity-reducing effect of processing

**Figure 6:**
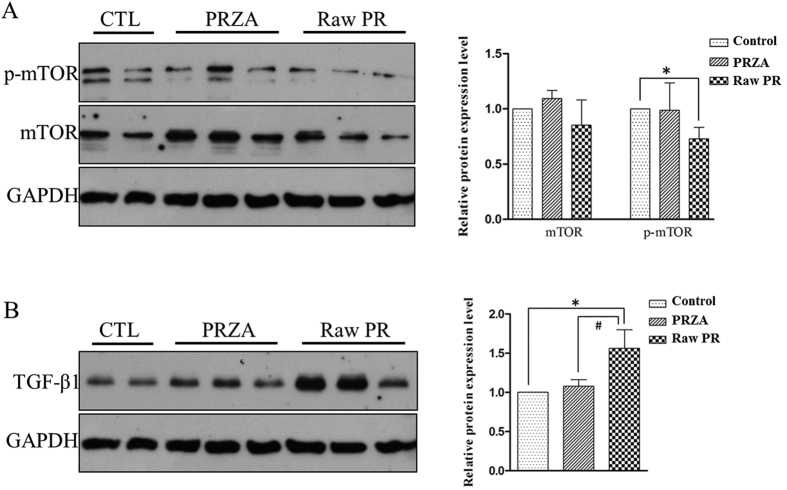
The protein expression levels in the heart tissues of vehicle-, raw PR- and PRZA-treated rats. (**A**) mTOR and phospho-mTOR protein expression levels; (**B**) TGF-β1 protein expression levels. The protein expression levels (left, representative results) were determined by Western blot analyses and relative band intensities (right) were analyzed by the Image J software. *p < 0.05, *vs*. control group; ^#^*p* < 0.05 *vs*. raw PR group.

In an attempt to verify the two IPA highlighted biological pathways (the mTOR and TGF-β signaling pathways) that potentially involved in raw PR-induced cardiotoxicity, we examined the expressions of the critical proteins in these two pathways in rat heart tissues. Western blot analyses showed that raw PR significantly decreased the phosphorylation of mTOR (the critical protein involved in mTOR signaling) compared to the vehicle (*p* < 0.05) (Fig. 6A). Previous studies have demonstrated that inhibition of mTOR signaling could induce cardiac diseases^23^,^24^. Hence, our data suggested that inhibition of mTOR signaling was one of mechanisms responsible for PR-induced cardiotoxicity. Meanwhile no significant difference in the mTOR phosphorylation was observed between control and PRZA groups (Fig. 6A), which supported our observations that processing reduced the cardiotoxicity of raw PR. In addition, raw PR dramatically increased the TGF-β1 (the critical protein involved in TGF-β signaling) protein expression level compared to the vehicle (*p* < 0.05), while, no significant difference in the protein expression level of TGF-β1 was observed between control and PRZA groups (Fig. 6B). Studies have demonstrated that up-regulation of the TGF-β1 expression could cause cardiac adverse effects^25^,^26^,^27^. Thus, it could be speculated that activation of the TGF-β signaling pathway contributed to raw PR-induced cardiotoxicity and that processing reduced the toxicity.

**Figure 7:**
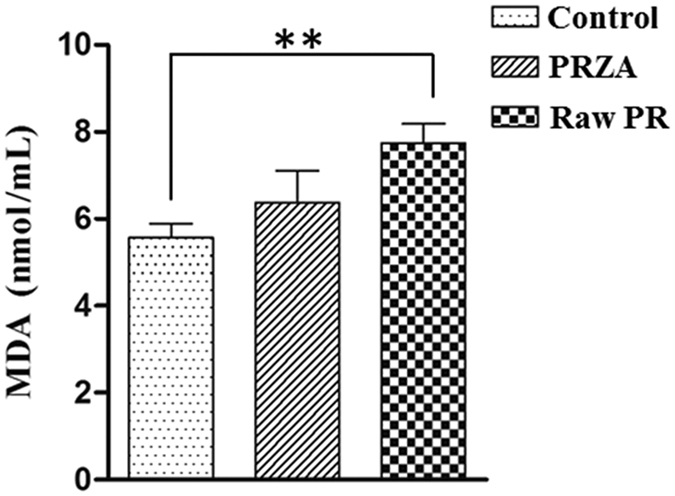
The MDA contents in the serum of vehicle-, raw PR- and PRZA-treated rats. **p < 0.01 *vs*. control.

To verify the predicted molecular network function, free radical scavenging that may involve in the toxicity-reducing effect of processing, we determined the MDA contents in the serum samples of vehicle-, raw PR- and PRZA-treated rats. MDA is a classic biomarker of oxidative stress, which is produced by free radicals in the body. MDA is widely used as an indicator of the free radical level^31^. As shown in Fig. 7, raw PR significantly increased the MDA content compared to the vehicle (*p* < 0.01), suggesting that raw PR caused oxidative stress in rats. While the MDA content in PRZA group was lower than that in raw PR group (*p* < 0.01), and no significant difference was observed in control and PRZA groups, suggesting that processing reduced the oxidative stress-causing effects of raw PR or processing enabled the herb to possess free radical scavenging property. These findings further suggest that free radical scavenging may be responsible for the cardiotoxicity-reducing effect of processing.

**Figure 8:**
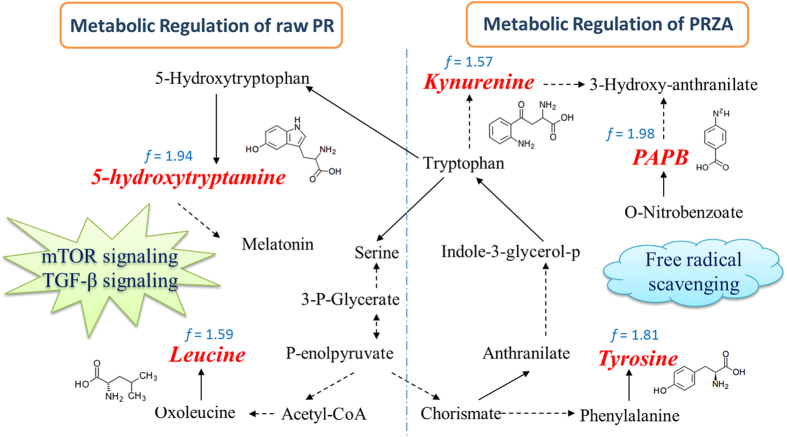
The characteristic metabolites and the regulated metabolic regulatory networks of raw PR and PRZA. Red italics molecules represent characteristic metabolites up-regulation in raw PR group and PRZA group; solid lines between molecules indicate a direct transforming relationship, while dotted lines indicate indirect relationships. “*f*” represents the fold change value of raw PR *vs*. control or PRZA *vs*. control.

Taken together, all above results suggest that inhibition of mTOR signaling and activation of the TGF-β pathway contributed to raw PR-induced cardiotoxicity, and free radical scavenging was, at least in part, responsible for the toxicity-reducing effect of processing. The characteristic metabolites and the metabolic regulatory networks of raw PR and PRZA were summarized in Fig. 8.

## Discussion

PR is toxic, it has irritant, teratogenic, carcinogenic, mutagenic, reproductive and organ toxicities. Many researchers have explored its organ toxicities, and found that PR could cause liver, kidney and heart toxicities. Intragastric administration of the water extract and the acid-water percolating liquid of PR could cause acute renal and liver damage and increase the activity of ALT and AST in mice^32^,^33^,^34^. In our experiments, we used the PR powder suspension to treat rats for 14 days, and found that raw PR caused heart damage. Some studies also have shown that PR powder suspension could cause heart damage^10^. The toxic chemical constituents and their contents may different in different herb extracts. Therefore, the toxicities of different extracts of an herb may be different in different animals even in one animal species. Whether the different extracts and different animals are the reasons for the observed different toxicities needs to be studied. PR is commonly used for treating cough, phlegm, vomiting and cancer. In the future, toxicity studies in the disease models can be conducted.

According to the TCM theory, processing can reduce the toxicity of the herbs. The theory has been supported by modern studies^35^,^36^. Many studies have shown that processing can reduce the toxicity of raw PR. People have studied the toxic components, toxicological effects and toxicokinetics of this herb^2^,^9^,^11^,^12^. To explore the toxicity-reducing mechanisms of processing, researchers have been studying the structural changes of toxic substances and the impact of the adjuvant materials used in processing^15^,^17^. However, the mechanisms of raw PR-induced toxicities and the toxicity-reducing effect of processing are still not fully understood. Therefore, we conducted metabonomics analyses to explore the mechanisms involved in raw PR-induced cardiotoxicity and the toxicity-reducing effect of processing.

A total of 10 significantly altered metabolites were identified in the serum samples of vehicle-, raw PR- and PRZA-treated rats (Table 1). Among them, three were anino acids. The observed changes in amino acid contents should be caused by the response of endogenous amino acid metabolism system, but not by the exogenous amino acid loading. In our experiments, we had fasted the rats for 24 h before blood sample collection. The half-lives of exogenous amino acids in rats are very short, for example, the half-lives of tryptophan and leucine are 4.34 h^37^ and 84.8 min^38^, respectively. Exogenous amino acids should be eliminated within 24 h, and could not be detected in the serum.

Two characteristic metabolites leucine and 5-HT were only up-regulated in raw PR group. Leucine, one of the branched-chain amino acids, is commonly catabolized in cardiac muscle and highly effective in activating mTOR signaling^21^,^22^. Some studies suggested that inhibition of mTOR signaling is closely related to the pathogenesis of cardiac hypertrophy^23^, which may cause heart failure, and lead to morbidity and mortality^39^,^40^; other studies demonstrated that inhibition of mTOR signaling induced acute cardiotoxicity^41^; our Western blot analyses showed that raw PR significantly decreased the phosphorylation of mTOR in heart tissues compared to the vehicle, suggesting that inhibition of mTOR signaling may contribute to raw PR-induced cardiotoxicity. However, in this study, leucine was up-regulated (Table 1), and the phosphorylation of mTOR was decreased (Fig. 6A) in raw PR group compared to control group, i.e., leucine up-regulation did not activate the mTOR signaling in heart tissues. This result is different from previous reports^21^,^22^. In our experimental setting, some raw PR chemicals-caused leucine up-regulation might activate mTOR signaling through some signaling pathways; whereas the effects of leucine on those pathways were probably opposite as expected because of the impact of other components of raw PR, resulting in the observed reduction of mTOR phosphorylation. Alternatively, a third group of compounds from raw PR might inhibit mTOR signaling by leucine-unrelated mechanisms in rat hearts. How mTOR phosphorylation in rat hearts was reduced in response to raw PR treatment is a question to be addressed. Inhibition of mTOR signaling is not necessarily due to the changes of leucine levels, although it may contribute to raw PR-induced cardiotoxicity.

Serotonin, also named as 5-HT, has growth-promoting effect on cardiac myocytes in physiological status^42^. While in pathological state, 5-HT plays a major role in the pathogenesis of the cardiac plaque formation and is considered as an etiologic agent in the development of heart diseases. Studies suggested that 5-HT up-regulation is related to the apoptosis of cardiomyocytes, which may lead to cardiac failure^43^. 5-HT up-regulation indeed causes cardiac hypertrophy, fibrosis, valvular and endocardial lesions^26^,^44^. Furthermore, 5-HT up-regulation elevates the TGF-β1 expression and activity, which may result in cardiac diseases^26^,^27^. As shown in Table 1, 5-HT was up-regulated in raw PR group compared to control group, and our Western blot analyses showed that raw PR significantly increased the expression level of TGF-β1 in heart tissues compared to the vehicle, suggesting that activation of the TGF-β signaling pathway also contribute to raw PR-induced cardiotoxicity. Based on the discovered toxicity mechanisms, people can improve the processing procedure of PR and can select its suitable combinational-use herbs in the clinic.

Previous study has demonstrated that the toxicity mechanisms of raw PR-induced toxicity and the toxicity-reducing effect of processing were related to inflammation^15^. Inhibition of mTOR signaling could induce inflammatory responses in cardiomyocytes^45^,^46^. In addition, studies have demonstrated that TGF-βs are often chronically overexpressed in disease states, such as cancer and inflammation^47^. Thus, it could be speculated that inhibition of mTOR signaling and activation of the TGF-β pathway contributed to raw PR-induced inflammation in heart tissues. Maybe that is the reason for our observed inflammatory-cell infiltration in the hearts of raw PR-treated rats.

Among the 10 significantly altered metabolites in the experimental groups, three metabolites kynurenine, PABA and tyrosine were only up-regulated in PRZA group. All these three metabolites have free radical scavenging properties^28^,^29^,^30^. Kynurenine is a metabolite of an amino acid tryptophan (a compound from fresh ginger^48^). It is a free-radical scavenger that is estimated to be at least 24 times more efficient than trolox for scavenging •OOH^28^,^49^. PABA, an intermediate in the synthesis of folate by many bacteria including Escherichia coli, is nontoxic and is regarded as a nutritional supplement that may alleviate fatigue, irritability, depression, weeping eczema, scleroderma, *etc*. Studies suggested that PABA prohibits the generation of free radicals by inhibiting the nitric oxide formation^29^. Additionally, several bioactive components from fresh ginger, such as 6-shogaol, zingerone and dehydrozingerone have potent free radical scavenging and tyrosinase inhibitory activities^30^,^50^,^51^. Tyrosinase is one of the important enzymes that involved in tyrosine degradation. Hence, tyrosine up-regulation may reflect the free radical scavenging capacity enhancement. Our results showed that raw PR significantly increased the MDA (an indicator of the free radical level) content compared to the vehicle, while the MDA content in PRZA group was lower than that in raw PR group, suggesting that processing enabled the herb to possess free radical scavenging property. Therefore, free radical scavenging may be responsible for the cardiotoxicity-reducing effect of processing.

Other five metabolites including KMTB, saccharopine, dihydrouracil, dhS1P and proline were identified to be altered in both raw PR and PRZA groups. Alterations of these metabolites might be responsible for the common toxic or therapeutic effects of raw PR and PRZA. Specifically, KMTB is a keto form of the sulfur-based amino acid methionine^52^,^53^. Measurement of KMTB has been considered as a potential means for detecting free radical generation *in vivo*. Previous studies suggested that the higher serum KMTB level, the more free radical generation^54^. In this study, KMTB was up-regulated in both raw PR and PRZA groups, up-regulation of KMTB may contribute to raw PR- and PRZA-induced oxidative damage. Saccharopine is an intermediate in the degradation of lysine in mammals. Saccharopine down-regulation might be due to the lysine reduction. Lysine plays a major role in calcium absorption, muscle protein building, as well as hormone, enzyme and antibody productions. Studies suggested that lysine deficiency causes immunodeficiency^55^,^56^. Saccharopine was down-regulated in both raw PR and PRZA groups, which might partially explain the toxicities of both raw PR and PRZA. Dihydrouracil is an intermediate in the catabolism of uracil (a nucleoside). Nucleosides are the important components of PR^57^. Dihydrouracil up-regulation should be attributed to the metabolism of raw PR and PRZA in the body. dhS1P is a bioactive lipid mediator that is similar to sphingosine-1 phosphate (S1P). Both dhS1P and S1P are catalyzed by sphingosine kinases. S1P regulates cardiac and vascular homeostasis^58^,^59^. It plays a causal role in the pathogenesis of many cardiovascular disorders, such as coronary artery disease, atherosclerosis, myocardial infarction, and heart failure^60^. dhS1P was up-regulated in both raw PR and PRZA groups, which might partially explain the toxicities of raw PR and PRZA. Proline, an α-amino acid, is regarded as a signaling molecule to modulate mitochondrial functions and trigger specific gene expressions^61^. It plays an important role in cellular homeostasis including redox balance and energy status^62^. Proline was up-regulated in both raw PR and PRZA groups, which might explain the common efficacies of both raw PR and PRZA.

In summary, we demonstrated that processing with ginger juice and alumen significantly reduced the cardiotoxicity of raw PR, which supported the TCM theory “processing can reduce the toxicity of raw PR”. Inhibition of mTOR signaling and activation of the TGF-β pathway contributed to raw PR-induced cardiotoxicity, and free radical scavenging might be responsible for the toxicity-reducing effect of processing, which shed new light on the mechanisms of raw PR-induced cardiotoxicity and the toxicity-reducing effect of processing with ginger juice and alumen. This study provides a scientific justification for the traditional processing theory, and should guide rational and safe clinical applications of PR by helping in optimizing its processing procedure and clinical compatibility. In addition, this study further suggests that metabolomics is a powerful approach for deciphering the complex mechanisms of herbal toxicities.

## Materials and Methods

### Chemicals and reagents

LC-MS grade acetonitrile, methanol and formic acid (FA) were purchased from RCI Labscan Ltd. (Thailand). Leucine enkephalin (spectroscopic grade) and all chemical standards were purchased from Sigma-Aldrich (MO, U.S.A.). Milli-Q water was prepared using a Milli-Q system (Millipore, MA, USA). Antibodies against phospho-AKT (Ser473), AKT, phospho-mTOR (Ser2448), mTOR and TGF-β1 were obtained from Cell Signaling Technology (Beverly, MA, USA). Anti-GAPDH was purchased from Santa Cruz Biotechnology (Santa Cruz, CA, USA). Goat anti-rabbit IgG, goat anti-mouse IgG and protein markers were supplied by Bio-Rad (Hercules, CA, USA).

### Herbal samples preparations

Raw PR (Supplementary Fig. 1), originated from Sichuan province (China), was provided by Kangmei Pharmaceutical Co. Ltd. Alumen was purchased from Jiangxi Zhangshu Tianqitang Traditional Chinese Medicine Co. Ltd. They were authenticated in accordance with the corresponding monograph in CP2010 (2010 edition of CP) by Dr. Hui Cao from Jinan University, China. Voucher specimens of raw PR (No. 20130510) and alumen (No. 1307007) were deposited at the School of Chinese Medicine, Hong Kong Baptist University.

Zingiberis Rhizoma Recens (fresh ginger) is the fresh rhizome of *Zingiber officinale* Rosc. It was collected from the market (originated from Shandong province, China) and authenticated in accordance with the monograph of Zingiberis Rhizoma Recens in CP2010 by the corresponding author. The contents of volatile oil and 6-gingerol in the fresh ginger were 0.29% (mL/g) and 0.07%, respectively (CP2010: not less than 0.12% and 0.05%, respectively).

Preparation of PRZA: soaked raw PR in water until the center of the cut surface was devoid of a dry core, boiled for 6 h after adding 12.5 kg alumen and 25 L freshly squeezed ginger juice for each 100 kg of raw PR, then took out and dried^63^.

Preparation of raw PR and PRZA suspensions: raw PR (200 g) or PRZA powder (200 g) was suspended in 600 mL 0.5% sodium carboxymethylcellulose (CMC-Na) solution to prepare 0.33 g/mL raw PR or PRZA suspension^9^,^10^. High performance liquid chromatography (HPLC) analysis showed that the contents of ephedrine in raw PR and PRZA were 2.93 μg/g and 1.27 μg/g, respectively (Supplementary Fig. 2). The representative positive BPI chromatograms of raw PR and PRZA were shown in Supplementary Fig. 3, and details of the MS of all components including ephedrine, 6-shogaol and gingerol corresponding to individual peaks were shown in Supplementary Table 1.

### Animals and treatments

A total of 18 male SD rats were obtained from the Chinese University of Hong Kong. They were reared under standard laboratory conditions (room temperature: 22–24 °C; relative humidity: 50–60%, a 12 h light/dark cycle), and fed with commercial food and tap water *ad libitum.* After one week of acclimatization, rats were randomly divided into three groups (n = 6), and daily intragastrically administered equal volume of the vehicle, raw PR or PRZA powder suspension at a dosage of 3 g/kg/day for 14 consecutive days^9^,^10^. The experimental protocol was approved by the Ethics Committee of Hong Kong Baptist University. Animal License (ID: (14–43) DH/HA&P/8/2/6 Pt.4.) was issued by the Department of Health of Hong Kong. All experimental procedures were conducted according to the principles expressed in the Declaration of Helsinki and the Guide for the Care and Use of Laboratory Animals published by the US National Institutes of Health. Every effort was made to reduce the number of animals used and minimize their pain and distress. The experimental design was shown in Supplementary Fig. 4.

### Animal sample preparation and analyses

Blood samples were collected from the retro-orbital venous plexus on day 15 and centrifuged at 3500 rpm for 10 min after standing for two hours at 4 °C. The serum was then transferred into new tubes and stored at −80 °C for further analysis. A portion of the collected serum was used for routine laboratory analyses of serum creatine kinase (CK), creatinine kinase-mb isoenzyme (CK-MB), lactate dehydrogenase (LDH), serum creatinine (SCR), blood urea nitrogen (BUN), aspartate aminotransferase (AST), alanine aminotransferase (ALT) and malondialdehyde (MDA) according to the manufacturer’s instructions of respective commercial test kits. Another portion of 200 μL of serum was added to 400 μL of acetonitrile, and the mixture was vortexed for 30 s. After centrifugation at 13,000 rpm for 10 min at 4 °C, the supernatant was pipetted out and lyophilized for LC-MS analyses. All rats were sacrificed following blood collection. Fresh cardiac, hepatic and renal tissues were obtained and fixed in 10% neutral buffered formaldehyde at 4 °C for paraffin embedment. Organ samples (4 μm) were sectioned and stained with H&E, and finally examined by light microscopy.

### LC-TOF-MS analyses

LC-TOF-MS analyses were performed by using an Agilent-1200 LC system coupled with an electrospray ionization (ESI) source (Agilent Technologies, Palo Alto, CA, USA) and an Agilent-6540 Q-TOF mass spectrometer. Separation of all samples were performed on an ACQUITY UPLC T_3_ C_18_ column (2.1 mm × 100 mm I.D., 1.8 μm) with a column temperature set at 35 °C. The flow rate was 0.45 mL/min, and the mobile phase was composed of 0.1% FA in acetonitrile and 0.1% FA in water. The following gradient program was used: 1% acetonitrile for 0–1 min; 1–70% acetonitrile for 1–10 min; 70–99% acetonitrile for 10–11 min; 99% acetonitrile for 11–13 min; re-equilibration step for 4 min. The sample injection volume was 2 μL.

Mass detection was operated in both positive and negative ion modes with the following setting: drying gas (N_2_) flow rate, 8 L/min; gas temperature, 350 °C; pressure of nebulizer gas, 35 psig; capillary voltage, 4500 V; fragmentor, 145 V; skimmer voltage, 65 V; scan range, m/z 50–1400. All analyses were acquired using the instrument mass spray to ensure accuracy and reproducibility. Leucine enkephalin was used as the instrument reference mass (m/z 556.2771) at a concentration of 50 fmol/μL with the flow rate 40 μL/min. Data profile was recorded at a speed of 0.15 s/scan and the scanning delay of 0.01s during analysis.

### Sequence analysis

The pooled quality control (QC) sample was analyzed at the beginning, the end, and randomly through the analytical run to monitor the stability of sequence analysis. The typical batch sequence of serum samples consisting of consecutive analysis cycles of 1 QC serum sample (at the beginning of the study) follow by 6 unknown serum samples. Meanwhile, samples were analyzed in a random order for a normal good practice. An identical sequence was repeated to complete the total set of injections (n = 22, including QCs) analyzed in less than 1 day per mode^64^,^65^.

### LC-MS data processing

The LC-MS raw data were exported by Agilent Mass Hunter Qualitative Analysis Software (Agilent Technologies, Palo Alto, CA, USA). The data of each sample were normalized to the total area to correct for the MS response shift between injections due to any possible intra- and inter-day variations. The sum of the ion peak areas within each sample was normalized to 10000. Multivariate analysis was performed using the Mass Profiler Professional (MPP) software B.12.00 (Agilent Technologies, Palo Alto, CA, USA). PCA analysis was used for classification of the differential metabolic phenotypes, and PLS-DA analysis was used for identify the differential metabolites.

### Pathway and network predication by the ingenuity pathway analysis (IPA)

The KEGG compound numbers and the matched identified metabolites in raw PR and PRZA groups were set up as the identifiers of two datasets. Each dataset was uploaded into the IPA system (http://www.ingenuity.com), which enables the discovery, visualization and exploration of molecular interactions to identify the biological mechanisms, pathways and functions most relevant to the experimental datasets. The proof-of-knowledge based IPA can be used to find out the biomarkers, and further predict treatment-affected biological pathways and networks based on these biomarkers. We utilized IPA “metabolic analysis” module to analyze the identified metabolites in raw PR and PRZA groups; and comparisons of the canonical pathways/biological networks between the two groups were carried out using the “comparison” module in IPA software. The scores associated with the canonical pathways analyses were calculated using logarithm of the *p*-value (*Fisher’s exact test*). Significances for biological functions were then assigned to each network by determining a *p*-value for the enrichment of the metabolites in the network for such functions compared with the whole Ingenuity Pathway Knowledge Base as a reference set.

### Western blot analyses

Western blot analyses of total protein lysates prepared from the heart tissues of control, raw PR and PRZA groups were performed. Protein concentrations were determined according to the Bio-Rad protein assay reagent. The heart tissue lysates were separated on 8% or 10% gels and transferred to nitrocellulose membranes. The membranes were incubated in 5% skim milk in TBS-T buffer at room temperature. Blocked membranes were incubated with primary antibodies at 4 °C overnight, followed by incubation with secondary antibodies at room temperature for 1 h. After washing in TBS-T, immunereactive bands were visualized by the chemiluminescence substrate (Thermo Scientific, Rockford, IL, USA).

### Statistical analysis

Results of body weights, biochemical assays and Western blot analyses were presented as mean ± SD. These data were analyzed by one-way ANOVA followed by the Dunnett’s multiple comparisons using GraphPad Prism version 5.0 (GraphPad Software, San Diego, CA, USA). *p* < 0.05 was considered statistically significant.

## Additional Information

**How to cite this article**: Su, T. *et al*. Metabolomics reveals the mechanisms for the cardiotoxicity of Pinelliae Rhizoma and the toxicity-reducing effect of processing. *Sci. Rep.* **6**, 34692; doi: 10.1038/srep34692 (2016).

## Supplementary Material

Supplementary Information

## Figures and Tables

**Table 1:** Significantly altered metabolites in the serum samples of vehicle-, raw PR- and PRZA-treated rats.

No.	t_R_ (min)	Extract mass	Formula	Compound	Fold change	
					Raw PR *vs.* Control	PRZA *vs.* Control
1	0.6906	115.0633	C_5_H_9_NO_2_	Proline	↑/1.87	↑/1.83
2	1.3981	131.0946	C_6_H_13_NO_2_	Leucine	↑/1.59	—
3	5.2621	208.0848	C_10_H_12_N_2_O_3_	Kynurenine	—	↑/1.57
4	1.7704	176.0950	C_10_H_12_N_2_O	5-hydroxytryptamine (5-HT)	↑/1.94	—
5	9.1083	114.0429	C_4_H_6_N_2_O_2_	Dihydrouracil	↑/1.92	↑/1.94
6	9.5202	381.2644	C_18_H_40_NO_5_P	Dihydrosphingosine 1-phosphate (dhS1P)	↑/1.62	↑/1.66
7	10.8046	148.0190	C_5_H_8_O_3_S	2-keto-4-methylthiobutyric acid (KMTB)	↑/1.44	↑/1.60
8	11.7082	276.1321	C_11_H_20_N_2_O_6_	Saccharopine	↓/−1.76	↓/−1.98
9	0.6774	137.0477	C_7_H_7_NO_2_	P-aminobenzoate (PABA)	—	↑/1.98
10	1.1796	181.0739	C_9_H_11_NO_3_	Tyrosine	—	↑/1.81

↑up-regulation; ↓down-regulation; —: no significant change.
