# Supplementary material for: Metabolomics reveals the mechanisms for the cardiotoxicity of Pinelliae Rhizoma and the toxicity-reducing effect of processing
Source: Sci Rep. 2016 Oct 4;6:34692. doi: 10.1038/srep34692 (PMC5048190; doi:10.1038/srep34692)
Supplement: Supplementary Information [file srep34692-s1.doc]

**Metabolomics reveals the mechanisms for the cardiotoxicity of Pinelliae Rhizoma and the toxicity-reducing effect of processing**

Tao Su^1^, Yong Tan^2^, Man-Shan Tsui^3^, Hua Yi^4^, Xiu-Qiong Fu^1^, Ting Li^1^, Chi-Leung Chan^1^, Hui Guo^1^, Ya-Xi Li^1^, Pei-Li Zhu^1^, Anfernee Kai-Wing Tse^1,5^, Hui Cao^6^, Ai-Ping Lu^1,5*^, Zhi-Ling Yu^1,5*^


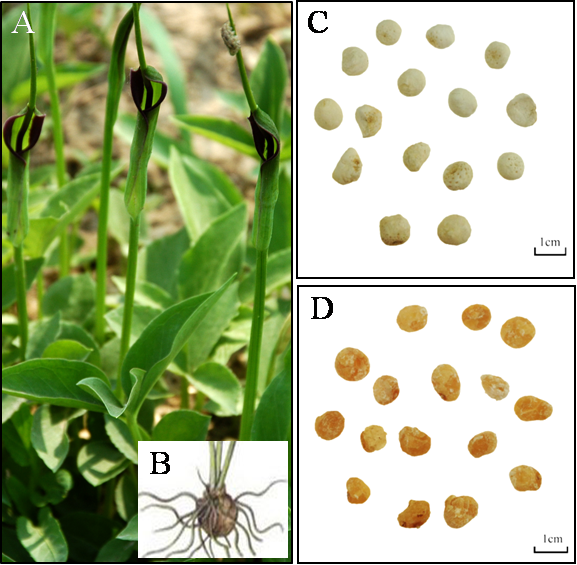


**Supplementary Fig. 1.** Pictures of the plant and tuber of *Pinellia ternata*, raw PR and PRZA samples. (A) *Pinellia ternata* (Photographed by Professor Hubiao Chen, School of Chinese Medicine, Hong Kong Baptist University); (B) tuber of *Pinellia ternata*; (C) Raw PR; (D) PRZA.


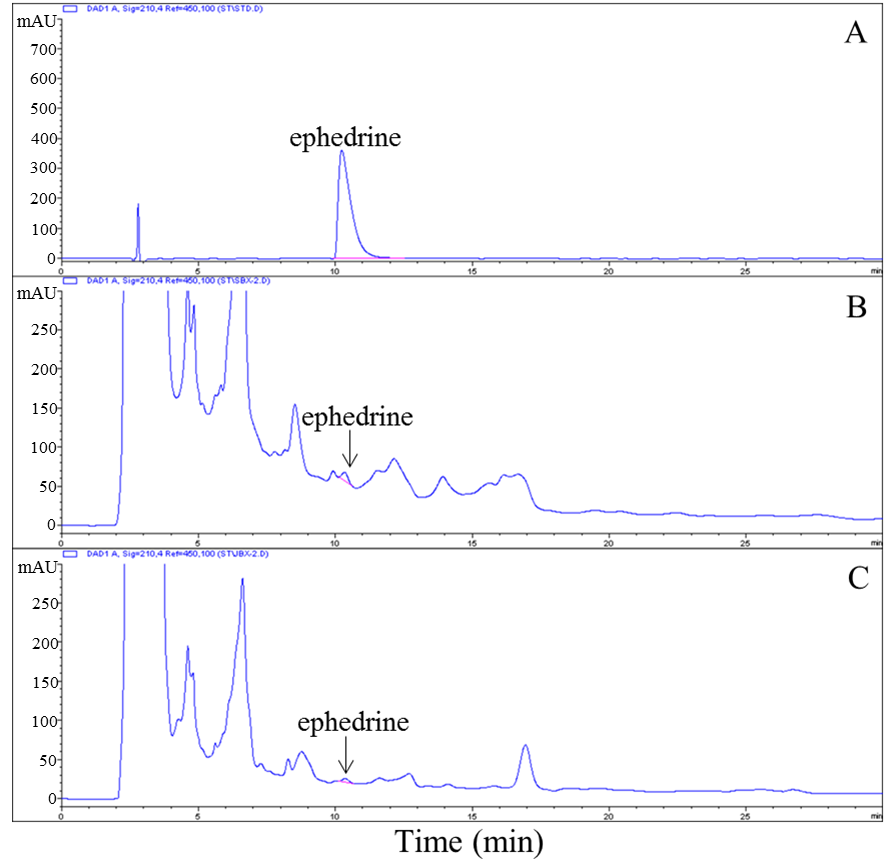


**Supplementary Fig. 2.** HPLC chromatograms of standard (ephedrine), raw PR and PRZA extracts. (A) standard; (B) raw PR; (C) PRZA. HPLC analyses were performed to control the quality of raw PR and PRZA extracts by using an Agilent1200 system (Agilent Technologies, Palo Alto, CA, USA) coupled with a Grace Alltima C_18_ column (4.6 mm×250 mm, 5μm) maintained at 25℃. The mobile phase was composed of C (0.08% [triethylamine](javascript:void(0);) in water) and D (methanol), and a gradient elution of 18% C and 82% D for 30 min was employed. The flow rate was 1 mL/min, the injection volume was 10 μL, and the detection wavelength was 210 nm. The HPLC analyses showed that the contents of [ephedrine](javascript:void(0);) in raw PR and PRZA were 2.93 μg/g and 1.27 μg/g, respectively.


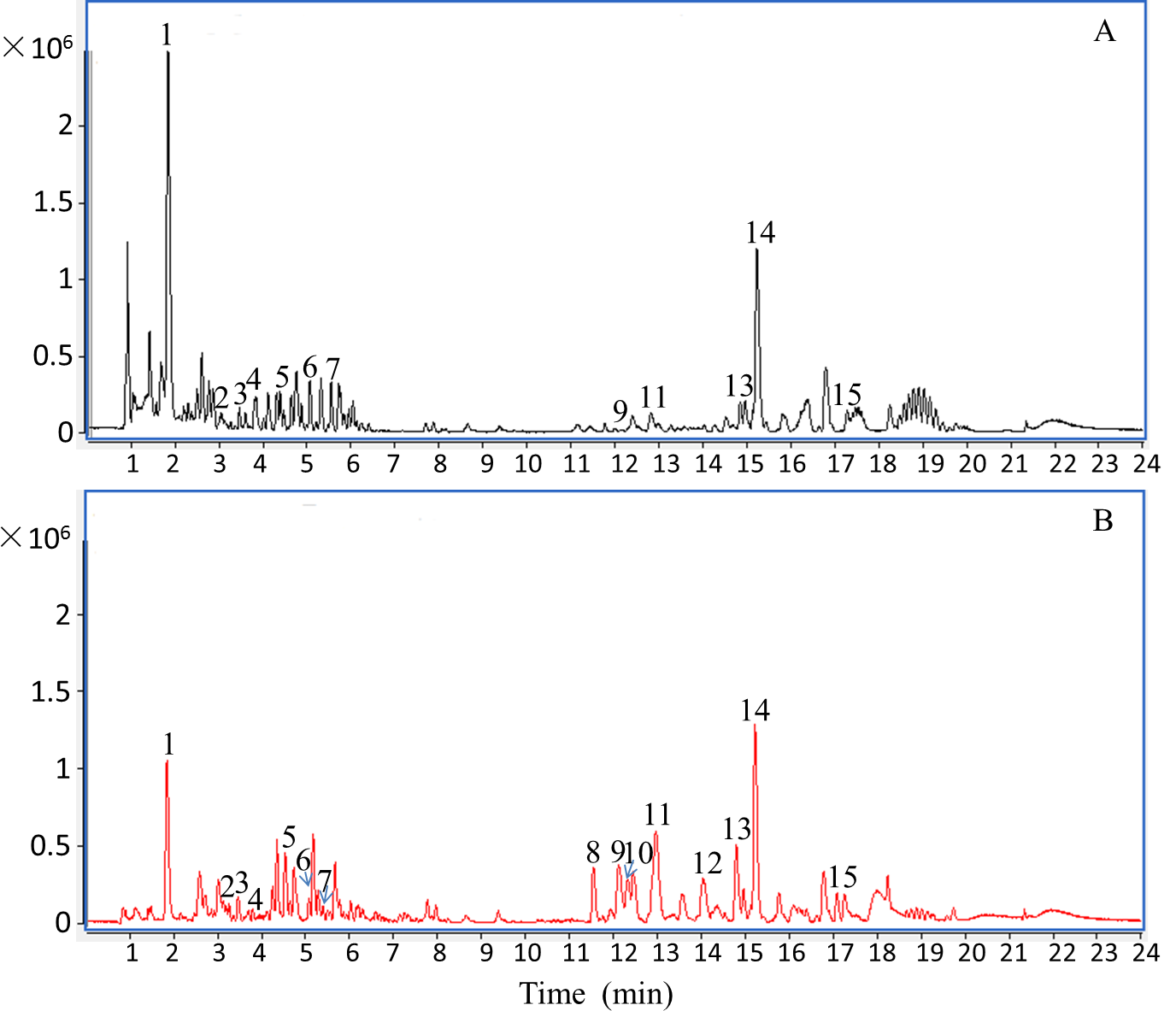


**Supplementary Fig. 3.** The representative positive base peak intensity chromatograms of raw PR and PRZA extracts. (A) Raw PR; (C) PRZA.


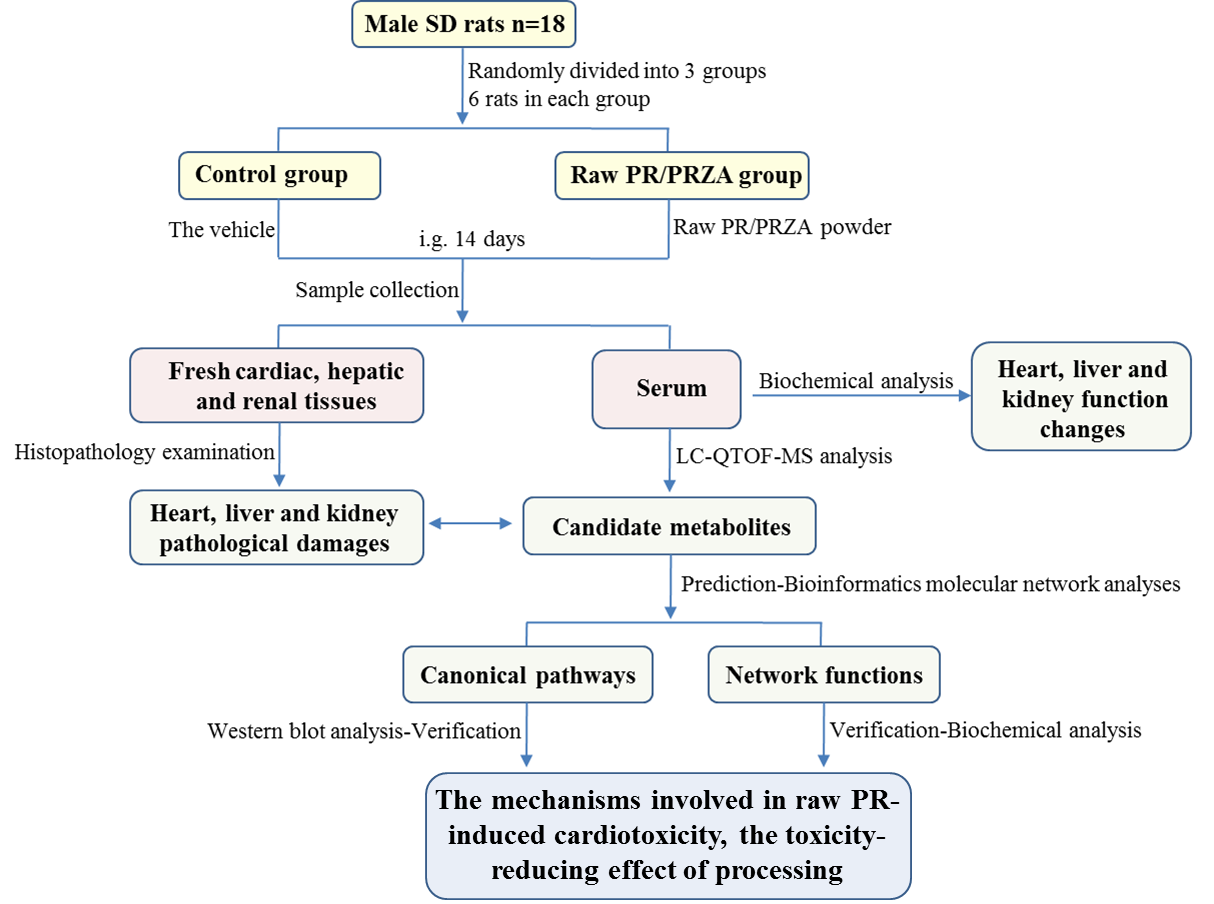


**Supplementary Fig. 4.** The experimental design.


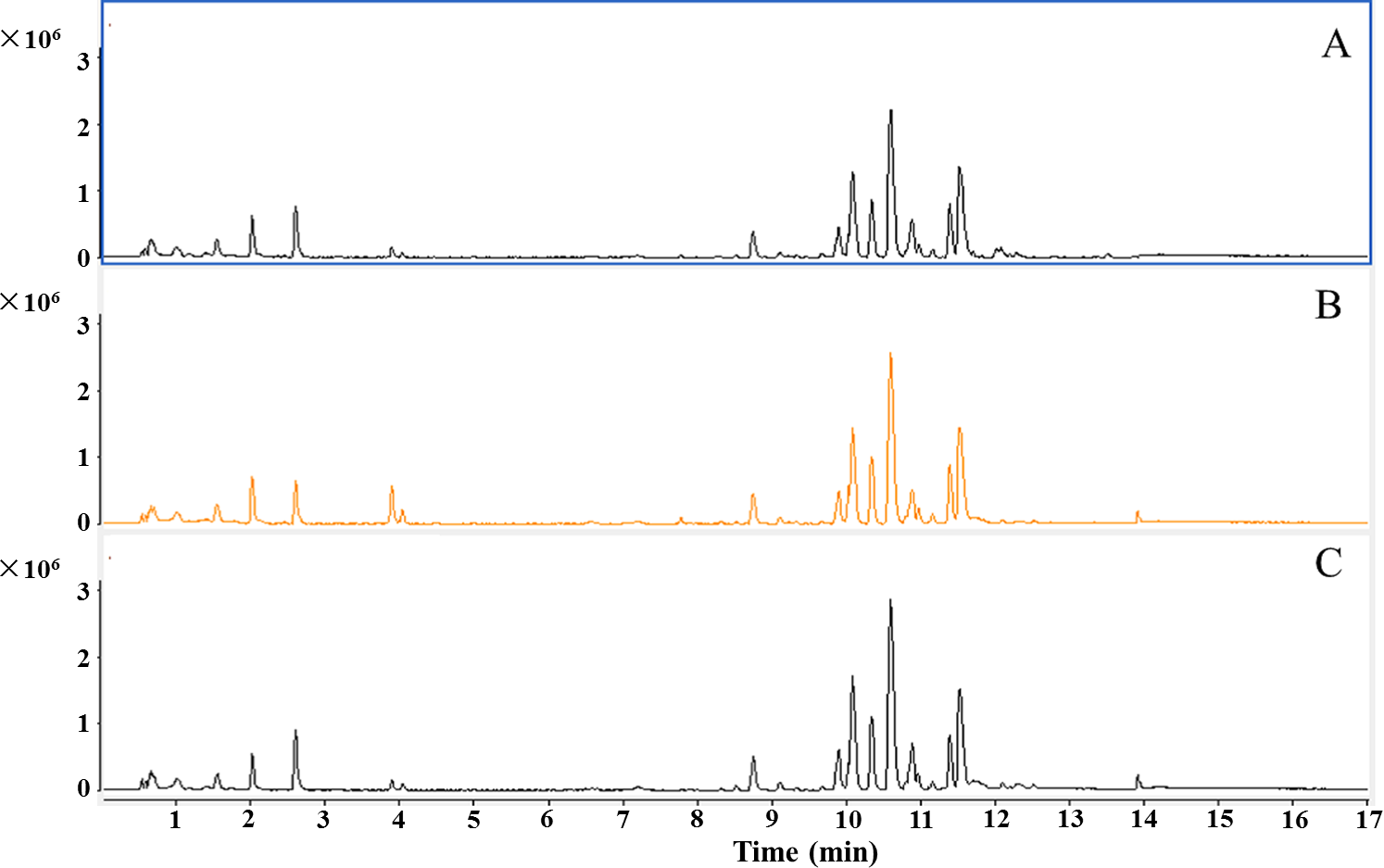


**Supplementary Fig. 5.** The representative positive base peak intensity chromatograms of the serum samples of vehicle-, raw PR- and PRZA-treated rats. (A) Control group; (B) Raw PR group; (C) PRZA group.

**
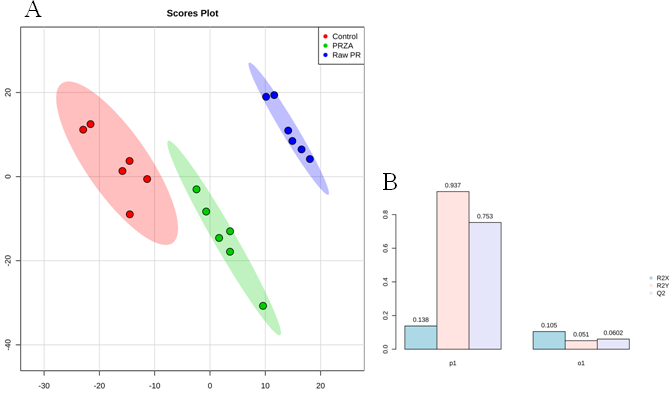
**

**Supplementary Fig. 6**. Results of the multiple pattern recognition of metabolites in control, raw PR and PRZA groups at the time point of day 14 (A). (red
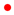
) Control group, (blue
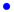
) Raw PR group, (green
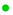
) PRZA group. PLS-DA scores plot (R^2^X=0.138, R^2^Y=0.937, and Q^2^=0.753) (B).

Supplementary Table 1. Compounds identified from raw PR and PRZA.

| Peak no. | t_R_ (min) | Assigned identity | Molecular | Mean measured mass (Da) | Mass accuracy (ppm) | Theoretical exact mass (Da) | Quasi-  molecular ion | Change trend after processing |
| --- | --- | --- | --- | --- | --- | --- | --- | --- |
| 1 | 1.848 | Ephedrine | C_10_H_15_NO | 166.0725 | 0.23 | 165.1154 | [M+H]^+^ | ↓^**^ |
| 2 | 3.058 | Ergosterol | C_28_H_44_O | 397.2444 | 1.01 | 396.3392 | [M+H]^+^ | ↓^*^ |
| 3 | 3.266 | NI | C_16_H_32_O_3_ | 295.1619 | 3.16 | 272.2351 | [M+Na]^+^ | ↓ |
| 4 | 3.808 | NI | C_24_H_37_N_3_O_4_ | 432.2800 | 1.73 | 431.2784 | [M+H]^+^ | ↓^**^ |
| 5 | 4.544 | NI | C_23_H_34_N_14_O_3_ | 555.3010 | 0.32 | 554.2938 | [M+H]^+^ | ↑^**^ |
| 6 | 5.068 | NI | C_47_H_41_NO_2_ | 652.4096 | 3.16 | 651.3137 | [M+H]^+^ | ↓^**^ |
| 7 | 5.552 | NI | C_20_H_51_N_9_O_10_ | 578.3756 | 0.87 | 577.3759 | [M+H]^+^ | ↓^**^ |
| 8 | 11.557 | 6-shogaol | C_17_ H_24_ O_3_ | 277.1820 | 2.54 | 276.1725 | [M+H]^+^ | ↑^**^ |
| 9 | 12.151 | Gingerol | C_17_H_26_O_4_ | 362.3061 | 1.21 | 294.1831 | [M+HCOONa]^+^ | ↑^**^ |
| 10 | 12.349 | NI | C_17_H_39_N_5_O_3_ | 362.3103 | 4.87 | 361.3053 | [M+H]^+^ | ↑^**^ |
| 11 | 12.967 | N,2-dimethyl-3-hydroxy-6-(9-phenylnonyl) piperidine | C_22_H_37_NO | 332.2999 | 3.21 | 331.2875 | [M+H]^+^ | ↑^**^ |
| 12 | 14.036 | NI | C_16_H_30_O_11_ | 399.1864 | -0.02 | 398.1788 | [M+H]^+^ | ↑^**^ |
| 13 | 14.791 | NI | C_8_H_8_O_2_ | 137.0619 | 1.99 | 136.0524 | [M+H]^+^ | ↑** |
| 14 | 15.245 | NI | C_22_H_45_N7O_7_ | 520.3482 | 0.91 | 519.3380 | [M+H]^+^ | ↑ |
| 15 | 17.279 | Paracoumaryl alcohol | C_9_H_10_O_2_ | 301.1432 | -1.87 | 150.1745 | [2M+H]^+^ | ↑ |

**p*＜0.05, ***p*＜0.01 *vs.* Raw PR; ↑ increased after processing; ↓ decreased after processing; NI: unidentified.
